# Ethnic inequalities in treatment with clozapine

**DOI:** 10.1192/j.eurpsy.2022.1565

**Published:** 2022-09-01

**Authors:** D. Fonseca De Freitas, I. Patel, G. Kadra-Scalzo, M. Pritchard, H. Shetty, M. Broadbent, R. Patel, J. Downs, A. Segev, M. Khondoker, J. Maccabe, K. Bhui, R. Hayes

**Affiliations:** 1 King’s College London & University of Oxford College London, Psychological Medicine & Department Of Psychiatry And Nuffield Department Of Primary Care Health Sciences, London, United Kingdom; 2 Institute of Psychiatry, Psychology, and Neuroscience, King’s College London, Department Of Psychological Medicine, London, United Kingdom; 3 South London and Maudsley NHS Foundation Trust, Maudsley Biomedical Research Centre, London, United Kingdom; 4 King’s College London, Academic Psychiatry, London, United Kingdom; 5 Tel Aviv University, Sackler Faculty Of Medicine, Tel Aviv-Yafo , Israel; 6 University of East Anglia, Norwich Medical School, Norwich, United Kingdom; 7 Universiy of Oxford, Dept Of Psychiatry And Nuffield Dept Of Primary Care Health Sciences, Oxford, United Kingdom

**Keywords:** clozapine, health inequalities, ethnicity, refractory psychosis

## Abstract

**Introduction:**

Ethnic disparities in treatment with clozapine, the antipsychotic recommended for treatment-resistant schizophrenia (TRS), have been reported. However, these investigations frequently suffer from potential residual confounding. For example, few studies have restricted the analyses to TRS samples and none has controlled for benign ethnic neutropenia.

**Objectives:**

This study investigated if service-users’ ethnicity influenced clozapine prescription in a cohort of people with TRS.

**Methods:**

Information from the clinical records of South London and Maudsley NHS Trust was used to identify a cohort of service-users with TRS between 2007 and 2017. In this cohort, we used logistic regression to investigate any association between ethnicity and clozapine prescription while adjusting for potential confounding variables, including sociodemographic factors, psychiatric multimorbidity, substance use, benign ethnic neutropenia, and inpatient and outpatient care received.

**Results:**

We identified 2239 cases that met the criteria for TRS. Results show that after adjusting for confounding variables, people with Black African ethnicity had half the odds of being treated with clozapine and people with Black Caribbean or Other Black background had about two-thirds the odds of being treated with clozapine compared White British service-users. No disparities were observed regarding other ethnic groups, namely Other White background, South Asian, Other Asian, or any other ethnicity.

**Conclusions:**

There was evidence of inequities in care among Black ethnic groups with TRS. Interventions targeting barriers in access to healthcare are recommended.

**Disclosure:**

During the conduction of the study, DFdF, GKS, and RH received funds from the NIHR Maudsley Biomedical Research Centre. For other activities outside the submitted work, DFdF received research funding from the UK Department of Health and Social Care, Janss

